# Effects of obesity indices/GDM on the pregnancy outcomes in Chinese women: A retrospective cohort study

**DOI:** 10.3389/fendo.2022.1029978

**Published:** 2022-11-18

**Authors:** Zhimin Song, Yan Cheng, Tingting Li, Yongfang Fan, Qingying Zhang, Haidong Cheng

**Affiliations:** ^1^ Department of Obstetrics, Obstetrics and Gynecology Hospital, Fudan University, Shanghai, China; ^2^ Department of Obstetrics and Gynecology, Women’s Hospital, Zhejiang University School of Medicine, Hangzhou, Zhejiang, China

**Keywords:** obesity, gestational diabetes mellitus (GDM), abdominal circumference (AC), pregnancy outcomes, pre-pregnancy body mass index (BMI)

## Abstract

**Objective:**

To analyze pregnancy complications and outcomes of mothers with obesity or gestational diabetes mellitus (GDM).

**Methods:**

15065 mothers were categorized into four and three groups by pre-pregnancy body mass index (preBMI) and abdominal circumference (AC), respectively, or divided into GDM or non-GDM groups. Logistic regression analysis was utilized to identify independent factors associated with pregnancy complications and outcomes.

**Results:**

The overweight and obesity groups accounted for 16.0% and 4.0% of the total population, respectively. GDM incidence rate was 12.3%. The overweight and obesity groups (pre-pregnancy body mass index [preBMI] ≥ 24 kg/m^2^) were at higher risks for GDM, hypertensive disorders of pregnancy (HDP), gestational proteinuria, postpartum hemorrhage, preterm delivery, fetal malformation or stillbirth, neonatal asphyxia, large for gestational age (LGA), shoulder dystocia, and increased cesarean section rate. Similar results were obtained with AC grouping. GDM pregnant women had higher risks of HDP, preterm delivery, small for gestational age (SGA), LGA, and increased cesarean section rate.

**Conclusion:**

People with obesity had a higher risk of adverse pregnancy outcomes. The recommended preBMI is 19.2-22.7 kg/m^2^. The recommended AC at 11-13^+6^ gestational weeks is 74.0-84.0 cm, and that value in normal preBMI is 74.0-82.0 cm.

## Introduction

Obesity has become more common in women of reproductive age, with yearly increase in its incidence rate. In the USA, while the proportion of women aged 20-39 years with body mass index (BMI) > 30 kg/m^2^ was less than 10% during the 1970s, this proportion had risen to 15% around 1990 and more than 25% by mid-2000s (*P*<0.001) (1). From 1993 to 2015, the prevalence of overweight (including obesity) in women in China increased from 29.2% to 39.6%, and the prevalence of abdominal obesity (AO) increased from 30.1% to 54.4% (*P*<0.001) (2). By 2025, more than 21% of women in the world are predicted to become obesity (3). Overweight and obesity have negative effects on the human body, which are associated with multiple comorbidities, such as diabetes, coronary heart disease, hypertension, colorectal cancer, gallbladder cancer, pancreatic cancer, ovarian cancer and asthma (4). Besides their impacts on the general population, these conditions also greatly affect pregnant women. Recent evidence suggested that nearly a quarter (23.9%) of the risk of any pregnancy complications could be attributed to maternal overweight or obesity, such as preterm premature rupture of membranes (PPROM), cesarean delivery, postpartum hemorrhage, and preeclampsia (5).

Gestational diabetes mellitus (GDM), an intolerant state of carbohydrate and one of the fastest growing pregnancy complications, is the first occurrence or discovery of abnormal glucose metabolism during pregnancy (6). Women with GDM have higher risks of developing preeclampsia as well as diabetes (predominantly type 2 diabetes) later in life. Furthermore, the offsprings of women with GDM are at increasing risks of macrosomia, neonatal hypoglycemia, hyperbilirubinemia, shoulder dystocia, birth trauma, and stillbirth (7). Compared to BMI, maternal fat distribution has been suggested as a better predictor of obesity-related adverse pregnancy outcomes. Waist circumference (WC) also predicted obesity-related adverse pregnancy outcomes as well as or better than BMI (8, 9). However, there is no consensus on abdominal circumference (AC) during early pregnancy and pre-pregnancy body mass index (preBMI).

The international guidelines on gestational obesity are mostly based on the Caucasian characteristics. Considering differences in races, environments (lifestyles and diets), and economic development trends, prevention and management of gestational obesity need to be improved through large-scale research based on the Chinese population (10).

## Methods

### Study population

Our retrospective cohort included 15065 women with singleton pregnancy, who had complete records of prenatal care services and deliveries at the Obstetrics and Gynecology Hospital of Fudan University from January 1, 2017, to June 30, 2019. Patients with pre-gestational diabetes mellitus, severe medical complications, and cancers were excluded. All procedures in this study were approved by the Human Research Ethics Committee of the Obstetrics and Gynecology Hospital, Fudan University (11) (grant no. 41).

### Clinical characteristics

Clinical characteristics (including age, gravidity, parity, and weight) were registered by self-reporting at the first prenatal visit at 11-13^+6^ gestational weeks. Delivery data, such as pregnancy and childbirth complications, pregnancy outcomes, newborn information, etc., were registered in the hospital after delivery.

### Anthropometric measurements

All participants underwent a physical examination at 11-13^+6^ gestational weeks. Pre-pregnancy weight was registered by self-reporting, while height and AC were measured in the clinic and preBMI was calculated by weight (kg) divided by the square of body height (m). Before AC measurement, pregnant women were instructed to empty their bladder, lie on their back, straighten their legs, and use a soft ruler to measure the distance of the abdomen at navel level for one circle. The minimum circumference was recorded to the nearest 0.1 cm. Two trained nurses completed a training program and obtained anthropometric measurements.

### Pregnancy complications and outcomes

Postpartum hemorrhage was defined as the blood loss ≥ 500 mL within 24 hours after vaginal delivery or ≥ 1000 mL after cesarean section. Preterm delivery referred to births at 28 weeks to < 37 weeks of pregnancy. Neonatal asphyxia was defined as neonatal Apgar score 1 minute or 5 minutes ≤ 7 points. Small for gestational age (SGA) was defined as the birthweight < the 10^th^ weight percentile of infants at the same gestational age. Large for gestational age (LGA) was defined as the birthweight > the 90^th^ weight percentile of infants at the same gestational age. Intrahepatic cholestasis of pregnancy (ICP) was diagnosed when pregnant women had fasting serum total bile acid ≥ 10 µmol/L and skin pruritus during pregnancy. The normal blood glucose values during 75-g oral glucose tolerance test (75g-OGTT) and fasting after 1 hours and 2 hours are < 5.1 mmol/L, 10.0 mmol/L and 8.5 mmol/L, respectively. If any blood glucose level reached or exceeded these aforementioned criteria, GDM could be diagnosed. Hypertensive disorders of pregnancy (HDP) included gestational hypertension, preeclampsia, eclampsia, chronic hypertension with preeclampsia and pregnancy complicated with chronic hypertension. Proteinuria during pregnancy was defined as proteinuria ≥ 0.3 g per 24 hours with normal blood pressure during pregnancy. Shoulder dystocia was diagnosed when the fetal neck retracted and the fetal chin compressed the perineum after the delivery. Consequently, the delivery of the fetal shoulder was hindered, and the congenital malformation of the fetus must be eliminated.

### Statistical analysis

All analyses were performed using SPSS 24.0 software (SPSS Inc., Chicago, IL, USA). Continuous variables were presented as mean (standard deviation [SD]) and skewed variables were presented as median (interquartile range). The Chi-squared test was used to analyze categorical variables. One-way analysis of variance (ANOVA) was used to analyze the significant differences among the characteristics of the study participants at entry, according to their BMI level. According to the BMI classification for Chinese adults published by the China Obesity Working Group in 2001, mothers were categorized into four groups: low weight (BMI < 18.5 kg/m^2^) normal weight (18.5 kg/m^2^ ≤ BMI < 24 kg/m^2^), overweight (24 kg/m^2^ ≤ BMI < 28 kg/m^2^), and obesity (BMI ≥ 28 kg/m^2^) (12). Participants were divided into three groups according to the AC quartiles: Q1, low-AC (AC < 74.0 cm); Q2/Q3, normal-AC (AC of 74.0-86.0 cm); Q4, high-AC (AC > 86.0 cm). Participants were also divided into GDM or non-GDM groups. Logistic regression analysis was carried out to explore the independent predictors of pregnancy complications and outcomes. The level of significance was set at *P<*0.05.

## Results

The normal-weight, low-weight, overweight, and obesity groups accounted for 67.1% (n = 10106), 12.9% (n = 1951), 16.0% (n = 2406), and 4.0% (n = 602) of the total population of 15065 participants, respectively. There were significant differences in age, AC, parity, gravidity, and gestational week of delivery among the four BMI groups (*P*<0.001). Within the obesity group, GDM incidence rate increased with increasing severity of obesity, which was consistent with findings from previous reports. Significant differences in cesarean section, preterm delivery, neonatal asphyxia, fetal malformation or stillbirth, LGA, SGA, shoulder dystocia, gestational proteinuria, postpartum hemorrhage, and HDP were detected among pregnant women from different BMI groups (*P*<0.05). The incidence of shoulder dystocia, proteinuria during pregnancy, postpartum hemorrhage, HDP, LGA, and neonatal asphyxia increased with increasing BMI. On the contrary, SGA was more likely to occur in participants with lower BMI. There was no significant difference ICP (*P*>0.05; **Table 1**).

**Table 1:** Clinical characteristics of the four groups divided by preBMI*(n=15065).

preBMI	<18.5 n=1951(12.9)	18.5-24 n=10106(67.1)	24-28 n=2406(16.0)	≥28 n=602(4.0)	P value
Age (years)	29.6 ± 3.6	30.7 ± 3.8	31.5 ± 4.2	31.7 ± 3.9	<0.001
AC(cm)	71.0 ± 4.7	79.1 ± 6.0	90.7 ± 6.5	100.0 ± 7.8	<0.001
Gravidity (%)					<0.001
Primigravid	1238(58.3)	5384(53.3)	1108(46.1)	260(43.2)	
Multigravid	813(41.7)	4722(46.7)	1298(53.9)	342(56.8)	
Parity (%)					<0.001
Primipara	1589(81.4)	7599(75.2)	1696(70.5)	428(71.1)	
Multipara	362(18.6)	2507(24.8)	710(29.5)	174(28.9)	
Gestational week of delivery	38.9 ± 1.4	38.9 ± 1.4	38.8 ± 1.6	38.6 ± 1.6	<0.001
Cesarean section (%)	380(25.1)	2297(32.0)	701(43.8)	214(52.6)	<0.001
Malformation or stillbirth (%)	22(1.1)	122(1.2)	51(2.1)	9(1.5)	0.005
Shoulder dystocia (%)	28(2.1)	122(1.9)	47(3.8)	15(5.5)	<0.001
Gestational proteinuria (%)	63(3.2)	433(4.3)	117(4.9)	39(6.5)	0.003
GDM (%)	160(8.2)	1071(10.6)	463(19.2)	166(27.6)	<0.001
Postpartum hemorrhage (%)	41(2.1)	220(2.2)	72(3.0)	24(4.0)	0.004
ICP (%)	16(0.8)	59(0.6)	12(0.5)	5(0.8)	0.474
HDP (%)	88(4.5)	802(7.9)	356(14.8)	200(33.2)	<0.001
Preterm delivery (%)	76(3.9)	403(4.0)	154(6.4)	36(6.0)	<0.001
SGA (%)	155(7.9)	512(5.1)	93(3.9)	14(2.3)	<0.001
LGA (%)	94(4.8)	925(9.2)	342(14.2)	124(20.6)	<0.001
Neonatal asphyxia(%)	26(1.3)	150(1.5)	55(2.3)	17(2.8)	0.003

*Values are expressed as mean ± standard deviation or number (%). Shoulder dystocia (%) is in the natural labor population (n=9110) and Cesarean section (%) is in the primipara population (n=11312). GDM, gestational diabetes mellitus; HDP, hypertensive disorders of pregnancy; ICP, intrahepatic cholestasis of pregnancy; SGA, small for gestational age; LGA, large for gestational age; and preBMI, pre-pregnancy body mass index.

**Figure 1:**
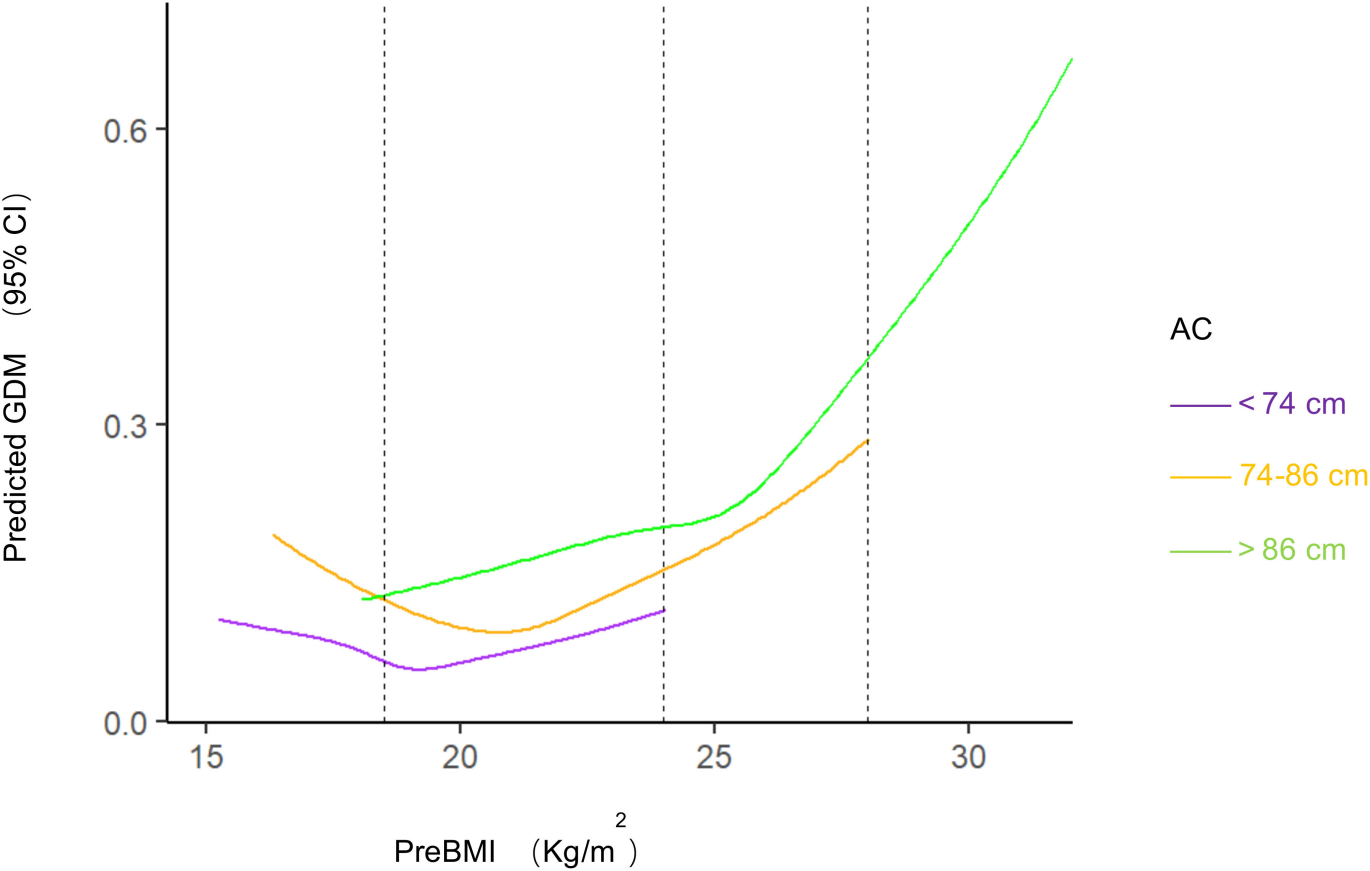
Predicted GDM with different BMI and AC. Restricted cubic splines to visualize the association between prepregnancy BMI and GDM stratified by abdominal circumference and estimates adjusted for age, gravidity, parity. Dashed vertical lines represent category thresholds of 18.5, 24.0 and 28.0 kg/m^2^ BMI, body mass index; AC, abdominal circumference; GDM, gestational diabetes mellitus.

There were significant differences in age, BMI, parity, gravidity, and gestational week of delivery among different AC groups (*P*<0.001; **Table 2**). Significant differences in GDM, cesarean section, preterm delivery, neonatal asphyxia, LGA, SGA, shoulder dystocia, gestational proteinuria, postpartum hemorrhage, and HDP were observed among pregnant women from different AC groups (*P*<0.05). The incidence of GDM, shoulder dystocia, proteinuria during pregnancy, postpartum hemorrhage, HDP, LGA, and neonatal asphyxia increased with increasing AC. SGA was more likely to occur in participants with lower AC. There was no significant difference in ICP and fetal malformation or stillbirth (*P*>0.05). In the low-AC group, preBMI was not significantly associated with GDM incidence (*P*>0.05). While preBMI and GDM incidence showed a U-shaped correlation in the normal-AC group (*P*<0.001), these parameters had a linear correlation (*P*<0.001) in the high-AC group (**Figure 1**).

**Table 2:** Clinical characteristics of the three groups divided by AC*(n=15065).

AC(cm)	Q1 (<74.0) n=3180	Q2/Q3 (74.0-86.0) n=8451	Q4 (>86.0) n=3434	P value
Age (years)	29.5 ± 3.5	30.8 ± 3.8	31.7 ± 4.1	<0.001
BMI	18.7 ± 1.4	21.2 ± 1.9	25.5 ± 2.9	<0.001
Gravidity (%)				<0.001
Primigravid	1981(62.3)	4395(52.0)	1514(44.1)	
Multigravid	1199(37.7)	4065(48.0)	1920(55.9)	
Parity (%)				<0.001
Primipara	2657(83.6)	6290(74.4)	2365(68.9)	
Multipara	523(16.4)	2161(24.8)	1069(31.1)	
Gestational week of delivery	39.0 ± 1.4	38.9 ± 1.4	38.7 ± 1.5	<0.001
Cesarean section (%)	615(24.3)	1986(33.3)	991(44.5)	<0.001
Malformation or stillbirth (%)	45(1.4)	102(1.2)	57(1.7)	0.145
Shoulder dystocia (%)	38(1.4)	105(2.0)	69(4.0)	<0.001
Gestational proteinuria (%)	108(3.4)	378(4.5)	166(4.8)	0.010
GDM (%)	207(6.5)	982(11.6)	671(19.5)	<0.001
Postpartum hemorrhage (%)	55(1.7)	203(2.4)	99(2.9)	0.008
ICP (%)	23(0.7)	53(0.6)	16(0.5)	0.381
HDP (%)	161(5.1)	713(8.4)	572(16.7)	<0.001
Preterm delivery (%)	104(3.3)	363(4.3)	202(5.9)	<0.001
SGA (%)	247(7.8)	399(4.7)	128(3.7)	<0.001
LGA (%)	145(4.6)	820(9.7)	520(15.1)	<0.001
Neonatal asphyxia (%)	44(1.4)	127(1.5)	77(2.2)	0.007

*Values are expressed as mean ± standard deviation or number (%);Shoulder dystocia (%) is in the natural labor population(n=9110) and Cesarean section (%) is in the primipara population (n=11312). AC, abdominal circumference; GDM, gestational diabetes mellitus; HDP, hypertensive disorders of pregnancy; ICP, intrahepatic cholestasis of pregnancy; SGA, small for gestational age; and LGA, large for gestational age.

Statistical analysis showed that the GDM incidence rate of the general population (n = 15065) was 12.3%. In the GDM (n = 1860) and non-GDM (n = 13205) groups, there were significant differences in age, preBMI, AC, parity, gravidity, gestational week of delivery, HDP, preterm delivery, cesarean section, LGA, and SGA (*P*<0.05). There was no significant difference in neonatal weight, proteinuria during pregnancy, ICP, postpartum hemorrhage, fetal malformation or stillbirth, neonatal asphyxia, and shoulder dystocia (*P>*0.05; **Table 3**).

**Table 3:** Clinical characteristics of the 2 groups divided by GDM*.

	GDM n=1860(12.3)	Control n=13205(87.7)	P value
Age (years)	32.0 ± 4.1	30.6 ± 3.8	<0.001
PreBMI	23.0 ± 3.6	21.5 ± 3.0	<0.001
Abdominal circumference(cm)	84.2 ± 9.4	80.2 ± 8.8	<0.001
Parity (%)			<0.001
Primipara	1312(70.5)	10000(75.7)	
Multipara	548(29.5)	3205(24.3)	
Gravidity (%)			<0.001
Primigravid	827(44.5)	7063(53.5)	
Multigravid	1033(55.5)	6142(46.5)	
Gestational week of delivery	38.6 ± 1.4	38.9 ± 1.5	<0.001
Neonatal weight(kg)	3.3 ± 0.5	3.3 ± 0.4	0.177
HDP (%)	276(14.8)	1170(8.9)	<0.001
Gestational proteinuria (%)	80(4.3)	572(4.3)	0.952
ICP (%)	14(0.8)	78(0.6)	0.401
Postpartum hemorrhage (%)	50(2.7)	307(2.3)	0.335
Malformation or stillbirth (%)	28(1.5)	176(1.3)	0.547
Cesarean section (%)	510(40.6)	3082(32.6)	<0.001
Preterm delivery (%)	105(5.6)	564(4.3)	0.007
SGA (%)	119(6.4)	655(5.0)	0.009
LGA (%)	222(11.9)	1263(9.6)	0.001
Neonatal asphyxia (%)	38(2.0)	210(1.6)	0.151
Shoulder dystocia (%)	29(2.9)	183(2.3)	0.215

*Values are expressed as mean ± standard deviation or number (%). Shoulder dystocia (%) is in the natural labor population (n=9110) and Cesarean section (%) is in the primipara population (n=11312). GDM, gestational diabetes mellitus; HDP, hypertensive disorders of pregnancy; SGA, small for gestational age; LGA, large for gestational age; and ICP, intrahepatic cholestasis of pregnancy.

Pregnancy complications and adverse pregnancy outcomes related to preBMI or AC included fetal malformation or stillbirth, shoulder dystocia, gestational proteinuria, GDM, postpartum hemorrhage, HDP, preterm delivery, LGA, SGA, and neonatal asphyxia (**Tables 1** and **2**). Logistic regression analysis showed that compared with pregnant women with normal preBMI, the overweight and obesity groups (preBMI ≥ 24 kg/m^2^) were at higher risks for GDM (odds ratio [OR]: 2.23; 95% confidence interval [CI]: 2.00-2.49), HDP (OR: 2.63; 95% CI: 2.34-2.69), gestational proteinuria (OR: 1.22; 95% CI: 1.01-1.47), postpartum hemorrhage (OR: 1.48; 95% CI: 1.16-1.89), preterm delivery (OR: 1.62; 95% CI: 1.36-1.94), fetal malformation or stillbirth (OR: 1.67; 95% CI: 1.22-2.28), neonatal asphyxia (OR: 1.63; 95% CI: 1.23-2.16), LGA (OR:1.82; 95% CI: 1.61-2.05), shoulder dystocia (OR: 1.81; 95% CI: 1.35-2.43), and increased cesarean section rate (OR: 1.72; 95% CI: 1.58-1.87). Compared with pregnant women with normal AC, the high-AC group (AC > 86.0 cm) were at higher risks for GDM (OR: 1.85; 95% CI: 1.66-2.06), HDP (OR: 2.17; 95% CI: 1.93-2.44), gestational proteinuria (OR: 1.09; 95% CI: 0.90-1.31), postpartum hemorrhage (OR: 1.21; 95% CI: 0.95-1.54), preterm delivery (OR: 1.39; 95% CI: 1.17-1.66), neonatal asphyxia (OR: 1.50; 95% CI: 1.13-2.00), LGA (OR: 1.66; 95% CI: 1.48-1.87), shoulder dystocia (OR: 2.01; 95% CI: 1.47-2.73), and increase cesarean section rate (OR: 1.61; 95% CI: 1.45-1.77) (**Table 4**).

**Table 4:** The relative risk of adverse pregnancy outcomes *.

	preBMI≥24 OR(95%CI)^a^	p value	GDM group OR(95%CI)^b^	p value	Q4 (>86.0 cm) OR(95%CI)^c^	p value
GDM	2.23(2.00-2.49)	<0.001			1.85(1.66-2.06)	<0.001
HDP	2.63(2.34-2.96)	<0.001	1.79(1.56-2.06)	<0.001	2.17(1.93-2.44)	<0.001
Gestational proteinuria	1.22(1.01-1.47)	0.036	0.99(0.78-1.26)	0.952	1.09(0.90-1.31)	0.393
Postpartum hemorrhage	1.48(1.16-1.89)	0.002	1.16(0.86-1.57)	0.335	1.21(0.95-1.54)	0.132
Preterm delivery	1.62(1.36-1.94)	<0.001	1.34(1.08-1.66)	0.007	1.39(1.17-1.66)	<0.001
Malformation or stillbirth	1.67(1.22-2.28)	0.001	1.13(0.76-1.70)	0.547	1.38(0.99-1.92)	0.052
Neonatal asphyxia	1.63(1.23-2.16)	0.001	1.29(0.91-1.83)	0.152	1.50(1.13-2.00)	0.005
SGA	0.69(0.56-0.86)	0.001	1.31(1.07-1.60)	0.009	0.78(0.64-0.96)	0.017
LGA	1.82(1.61-2.05)	<0.001	1.28(1.10-1.49)	0.001	1.66(1.48-1.87)	<0.001
Shoulder dystocia	1.81(1.35-2.43)	<0.001	1.03(0.69-1.52)	0.903	2.01(1.47-2.73)	0.005
Cesarean section	1.72(1.58-1.87)	<0.001	1.41(1.28-1.56)	<0.001	1.61(1.45-1.77)	<0.001

*OR, odds ratio; GDM, gestational diabetes mellitus; HDP, hypertensive disorders of pregnancy; SGA, small for gestational age; LGA, large for gestational age; preBMI, pre-pregnancy body mass index; AC, abdominal circumference. Shoulder dystocia (%) is in the natural labor population (n=9110); Cesarean section (%) is in the primipara population (n=11312). a: relative risk of adverse pregnancy outcomes between the overweight + obesity group (preBMI≥24) and the normal weight group; b: relative risk of adverse pregnancy outcomes between the GDM and non-GDM groups; c: relative risk of adverse pregnancy outcomes was compared between the normal AC and high-AC groups.

GDM-related pregnancy complications and adverse pregnancy outcomes included HDP, preterm delivery, LGA, and SGA (**Table 3**). Logistic regression analysis showed that compared with non-GDM pregnant women, pregnant women with GDM had higher risks of HDP (OR: 1.79; 95% CI: 1.56-2.06), preterm delivery (OR: 1.34; 95% CI: 1.08-1.66), SGA (OR: 1.31; 95% CI: 1.07-1.60), LGA (OR: 1.28; 95% CI: 1.10-1.49), and increased cesarean section rate (OR: 1.41; 95% CI: 1.28-1.56) (**Table 4**).

According to the aforementioned data, 6113 pregnant women with obesity-related delivery complications and adverse pregnancy outcomes were excluded, and the remaining sample size was 8952. By analyzing the preBMI quartile range of 8952 pregnant women without BMI-related adverse pregnancy outcomes, the recommended preBMI is 19.2-22.7 kg/m^2^. Similarly, the recommended AC at the time of card establishment is 74.0-84.0 cm, and this value for normal preBMI in pregnant women without BMI-related adverse pregnancy outcomes (n = 6300) is 74.0-82.0 cm (**Table 5**).

**Table 5:** Recommended obesity indicators*.

Percentile	preBMI^a^	AC(cm)^a^	AC(cm)^b^
5	17.4	68.0	70.0
10	18.0	70.0	72.0
25	19.2	74.0	74.0
50	20.7	78.0	78.0
75	22.7	84.0	82.0
90	24.8	91.0	86.0
95	26.3	96.0	89.0

*a: recommended preBMI and AC in pregnant women without preBMI-related adverse pregnancy outcomes (n=8952); b: recommended AC in normal preBMI in pregnant women without preBMI-related adverse pregnancy outcomes (n=6300) preBMI, pre-pregnancy body mass index and AC, abdominal circumference.

## Discussion

### Main findings

Compared with pregnant women with normal preBMI, the overweight and obesity groups (preBMI ≥ 24 kg/m^2^) were at higher risks for GDM, gestational proteinuria, postpartum hemorrhage, preterm delivery, fetal malformation or stillbirth, neonatal asphyxia, LGA, shoulder dystocia, and increased cesarean section rate, while their risk of SGA decreased. Pregnant women with GDM had higher risks of HDP, preterm delivery, SGA, LGA, and increased cesarean section rate.

### Strengths and limitations

This study represented a large-scale, population-based analysis with 30 months of data from a validated database. Information on maternal conditions and pregnancy outcomes was accurately captured in our database. However, this study had a single-center retrospective design, and some patients were not included in the study because of the lack of data. Additionally, increased early gestational AC in mothers with different gestational weeks might have affected the interpretation of the results. We could further explore the relationship between the onset of obesity and GDM for the Chinese population. As such, more targeted methods to prevent weight gain and GDM could be recommended, with a focus on the relationship between obesity and adverse pregnancy outcomes. Additional multi-center studies with large sample sizes are needed to generate more authoritative recommendations for pre-pregnancy indicators of obesity.

### Interpretation

Obesity has short-term and long-term adverse consequences for both mothers and children. Insulin resistance increases in early pregnancy, while glucose intolerance and fetal overgrowth occurs in late pregnancy. Overweight and obesity before pregnancy could increase the risk of gestational diabetes mellitus and cesarean section rate (13). A study in Croatia noted that pregnant women with obesity had a higher risk of preterm delivery, induced labor and cesarean section than normal pregnant women (14). Obesity before pregnancy and excessive weight gain during pregnancy could also lead to a higher risk of adverse pregnancy outcomes (15). Maternal obesity (OR: 2.16; 95% CI: 1.46-3.18) and GDM (OR: 2.21; 95% CI: 1.38-3.52) had independent effects on neonatal obesity (16). Newborns also had increased risks of long-term obesity, cardiovascular disease, and metabolic disease (17, 18). The hospital stay of the infants born to mothers with obesity was longer (3.9 days; *P*<0.005) *(* 19). Women with obesity had a higher risk of anesthesia difficulties and complications. In this regard, evaluation by anesthesiologists before labor and the use of appropriate doses of antibiotics before cesarean section to prevent thrombosis in pregnant women with obesity are recommended. The American College of Obstetricians and Gynecologists (ACOG) recommends that women at high risk of deep vein thrombosis (BMI ≥ 35 kg/m^2^, previous history of deep vein thrombosis, any thrombotic disease, etc.) should take mechanical and drug-induced thrombosis preventative measures while undergoing cesarian section (20, 21). The obstetric management of pregnant women with obesity should focus on the identification, treatment, and prevention of obesity-related complications (22). In this regard, women with BMI ≥ 25 kg/m^2^ are recommended to lose weight at a rate of no more than 0.5-1 kg per week before pregnancy and encouraged to lose at least 5% of their weight. The ACOG also recommends maintaining a daily healthy diet during pregnancy and exercising moderately for at least half an hour every day. This study employed a large sample size to determine the recommended preBMI, which could become a reference for the development of relevant guidelines.

Abdominal obesity in pregnant women has not received enough attention compared to BMI. Over a 23-year period, the age-standardized mean WC values showed a significant increase among Chinese adults with BMI < 25 kg/m^2^, with an increasing mean value from 74.0 cm to 78.5 cm (*P*<0.001). The mean WC and the prevalence of abdominal obesity among Chinese adults with normal BMI increased continuously from 1993 to 2015. Increasing trends were noted in both sexes, all age groups, rural and urban regions, all groups with different educational statuses (23), and pregnant women. Even in pregnant women with normal BMI, those with higher AC were more likely to develop GDM. Compared to BMI, maternal fat distribution has been suggested as a better predictor of obesity-related adverse pregnancy outcomes, and central adiposity in early to mid-pregnancy or, at the earliest, 365 days prior to conception could be a potential risk factor, in addition to BMI, for risk stratification of pregnant women (24). During the first trimester, AC of the pregnant woman does not increase significantly. We used AC at 11-13^+6^ gestational weeks to evaluate abdominal obesity, which had some limitations. However, there is no consensus on the recommended AC value in early pregnancy. Therefore, our study might serve as a reference and provide some insights for future studies.

Among the pregnant women with prenatal examination in our hospital, those with preBMI ≥ 24 kg/m^2^ or excessive weight gain during pregnancy were recommended to visit the nutrition clinic for diet and exercise guidance, and most pregnant women showed good compliance. In this study, using preBMI-related delivery complications and adverse pregnancy outcomes, the recommended preBMI is 19.2-22.7 kg/m^2^. The recommended AC at 11-13^+6^ gestational weeks is 74.0-84.0 cm, that value in normal preBMI is 74.0-82.0 cm.

GDM is associated with multiple delivery complications and perinatal outcomes, such as hypertriglyceridemia preterm rupture of membranes, preterm delivery, postpartum hemorrhage, fetal distress, etc. (*P*<0.05) (13). A prospective cohort study of 694 pregnant women in Ethiopia found that compared to normal pregnant women, those with GDM had a higher risk of adverse pregnancy outcomes (OR: 1.58; 95% CI: 1.22-2.04) and higher incidence of gestational hypertension, preterm rupture of membranes, prenatal hemorrhage, and postpartum hemorrhage (25). In an Australian single-center retrospective cohort study, the occurrence of GDM in pregnant women was associated with gestational hypertension, but did not affect adverse pregnancy outcomes, such as cesarean section, perineal incision rate, postpartum hemorrhage, and low birthweight (26). At the same time, a study in India found that under the guidance of the structured care model for Indian women, the perinatal outcomes of GDM patients, including cesarean section, preeclampsia, oligohydramnios or excessive amniotic fluid, preterm birth, neonatal death, fetal distress, hyperbilirubinemia and low birth weight, were similar to those of non-GDM patients (27). Our study did not analyze complications associated with postpartum depression, which is more common in obesity and GDM mothers. In this regard, the ACOG recommends that all mothers should screen for depression at least once postpartum.

Compared with most reports, improved GDM-related pregnancy outcomes were observed in our study. After 24-28 weeks of OGTT in our hospital, when GDM diagnosis was performed, diet and exercise guidance and close monitoring of blood sugar level at the outpatient clinic were recommended, and the patient’s compliance was improved. As such, the perinatal outcomes has been improved with current standardized management mode of GDM in our hospital.

## Conclusion

People with obesity had a higher risk of adverse pregnancy outcomes. Before pregnancy, BMI as well as the occurrence of abdominal obesity should be controlled. The recommended preBMI is 19.2-22.7 kg/m^2^. The recommended AC at 11-13^+6^ gestational weeks is 74.0-84.0 cm, and that value in normal preBMI is 74.0-82.0 cm.

## Data availability statement

The data analyzed in this study is subject to the following licenses/restrictions: The datasets generated and/or analysed during the current study are not publicly available due patient privacy but are available from the corresponding author on reasonable request. Requests to access these datasets should be directed to Haidong Cheng, Email: hdcheng_2003@163.com.

## Author contributions

HDC and QYZ designed the study. ZMS supervised the laboratory exams and data collection. ZMS, TTL and YFF analysed and interpreted the data. ZMS and YC wrote the first draft of the paper. HDC and QYZ edited the paper. All authors contributed to the article and approved the submitted version.

## Funding

This study was supported by the Fund of the National Natural Science Foundation of China (Grant No. 81471469 and Grant No. 81871184).

## Acknowledgments

We thank all the patients and their families for their cooperation and contribution.

## Conflict of interest

The authors declare that the research was conducted in the absence of any commercial or financial relationships that could be construed as a potential conflict of interest.

## Publisher’s note

All claims expressed in this article are solely those of the authors and do not necessarily represent those of their affiliated organizations, or those of the publisher, the editors and the reviewers. Any product that may be evaluated in this article, or claim that may be made by its manufacturer, is not guaranteed or endorsed by the publisher.
